# Assessing the educational performance of different Brazilian school cycles using data science methods

**DOI:** 10.1371/journal.pone.0248525

**Published:** 2021-03-17

**Authors:** Joyce de Souza Zanirato Maia, Ana Paula Arantes Bueno, João Ricardo Sato

**Affiliations:** 1 Centro de Engenharia, Modelagem e Ciências Sociais Aplicadas (CECS), Universidade Federal do ABC, São André, Brazil; 2 Centro de Matemática, Computação e Cognição (CMCC), Universidade Federal do ABC, São Bernardo do Campo, Brazil; University of Texas Southwestern Medical Center at Dallas, UNITED STATES

## Abstract

Educational indicators are metrics that assist in assessing the quality of the educational system. They are often associated with economic and social factors suggested to contribute to good school performance, however there is no consensus on the impact of these factors. The main objective of this work was to evaluate the factors related to school performance. Using a data set composed by Brazilian schools’ performance (IDEB), socioeconomic and school structure variables, we generated different models. The non-linear model predicted the best performance, measured by the error and determination coefficient metrics. The heterogeneity of the importance of the variable between school cycles and regions of the country was detected, this effect may contribute to the development of public educational policies.

## Introduction

Indicators are statistics that reveal the performance of the educational system. Although they are not perfect descriptors, they are critical for evaluating past policies and designing new strategies to improve results [1–3]. In addition, they are useful to compare school performance in different periods (temporal analysis) or between different locations (regional analysis). The Program for International Student Assessment (PISA) is the main educational indicator, held every three years by the Organization for Economic Cooperation and Development (OECD), which measures the performance of 15-year-old students enrolled in schools using mathematics, reading and science tests. This indicator has been pointed out as a superior measure to quantify the country´s economic development in contrast with the commonly used years of education [4]. Thus, for more detailed analysis, national indicators are potentially more effective to assess and evaluate the main aspects of the educational system.

Historically, the students’ socioeconomic status is often correlated with their performance [5–8], and considered as one of the most relevant conditions for successful school results [9, 10]. In the past few years, socioeconomic differences have increased dramatically in Brazil [11], therefore new results may complement previous findings and assist public decision makers to propose new alternatives to mitigate poor academic performance.

The Brazilian educational system is marked by the slow development of formal education, initially restricted to social layers with greater economic power, which has contributed to the economic disparities across the regions of the country [12, 13] S1 Fig and S1 Table. Several measures have been taken and school enrollment has increased massively in recent decades [12, 14], moving the focus now to difficulties related to quality and continuity of the studies until the final years of high school.

Therefore, measuring educational performance has become the main point, as the results allow to assess the efficiency of the educational system and constitute a new benchmark for changes in formulation and implementation of educational policies. As resources are limited, the indicators allow the identification of elements to be improved and are also a form of public transparency so society can verify if the resources allocated to Education are producing the expected results.

In Brazil, the Basic Education Development Index (IDEB) is the main descriptor of educational quality [15]. This indicator has shown improvement in recent years, however, not all schools followed this trend, making the educational performance in the different regions of the country very heterogeneous. It is unlikely that Brazil will reach its educational goal set for 2022, when all schools, regardless of school cycle, should obtain a minimum score of 6 points out of 10 on the IDEB scale.

The National Institute of Educational Studies and Research Anísio Teixeira (INEP) produces numerous data that could potentially help in the analysis of factors that may influence school performance and, therefore, help to formulate improvements in educational policies. However, those data are not fully explored and only a few studies use sophisticated econometric tools, generating macro insights on the educational level. Most studies apply traditional methods that have important limitations (e.g., the impossibility of including many descriptors in the models without loss of efficiency, the difficulty in dealing with a wide range of data). In general, the most studied factors are those with the greatest impact on academic performance: (i) socioeconomic situation of the family [5, 9, 16]; (ii) human capital of student´s parents [17]; (iii) grade repetition rate [18] and (iv) student’s personality [19, 20].

The study of such factors can benefit from the use of scientific data analysis techniques. Numerous research fields (e.g., health sciences, marketing, national security and others) have benefited from using different analysis techniques such as Machine Learning and Data Science [21, 22]. Several educational researches have been exploring those methodologies, focusing on school dropout, grade prediction, data analysis of online courses and others, but only a few with large-scale spatial data [23–25].

Considering the low educational performance, the models of multiple linear regression, penalized regression and gradient boosting machine (GBM), we have assessed an IDEB data set of different groups of descriptive variables to evaluate the predictive power of school performance, using variables that describe school structure and organization, in addition to socioeconomic variables. We have applied different analysis, including non-linear models. This study is expected to help in the conception of new perspectives for the design of educational policies.

## Methods

### Data

The public data set comes from different sources, all of them available on government websites with free access, partly related to education and partly related to socioeconomic information. The educational variables describe the performance of students in each school (IDEB) [15], the infrastructure of the school (School Census) and the performance indicators of teachers and school management (INEP) [14]. The socioeconomic variables bring to the model a perspective of monetary importance. Thus, the analysis included the average salary of teachers per municipality, educational Gross Domestic Product (GDP) per capita, and committed municipal expenditure, regarding the year of 2017. In this period, there were 5,570 municipalities in Brazil and 66,040 IDEB indicators available with other exploratory variables.

### Educational variables

IDEB is calculated every two years and is an index that simultaneously lists two fundamental characteristics in school evaluation: school performance and proficiency [14, 15] The calculation consists of the product of the average pass rate by the average proficiency of students in Portuguese language and Mathematics tests (SAEB), which are applied in the final years of each educational cycle, meaning in the 5th and 9th years of elementary school, and in the 3rd year of high school. Due to its periodicity, it is possible to assess the impact of short-term policies.

The descriptive variables of the school structure were obtained by the 2017 School Census [14]. The census is a survey of statistical data with broad objectives, and all educational institutions are required to participate in the research. The data are disseminated at different levels, which means that it is possible to analyze the information regarding enrollments, classes, teachers and schools. The variables selected from the census in the composition of the models are all binary, which indicates the presence or not of the resource. These variables in subsets allow the evaluation of four categories: Basic Structure (water, food, garbage collection, energy, sewer, adaptation to special needs); Environmental Diversity (complementary activities, auditorium, science laboratory, computer laboratory, sports court); School Structure (library, kitchen, principal’s office, municipal dependency, proportion of girls in school, cafeteria, school office, pedagogical support, teachers room, type of location: urban or rural) and Technology (computer, printer, internet access, TV) S2–S7 Tables.

Educational indicators are summarized variables generated by INEP using data from the School Census, with longitudinal monitoring and crossing information from other surveys. They are specific, so the perception of school characteristics is immediate. Therefore, these indicators were inserted in the studied models: Average Number of Daily Class Hours, Teaching Effort Index, Teaching Regularity Index, Management Complexity Index, Rate of Teachers with Higher Education, Average Number of Students per Classroom and Adequacy of Teacher Education.

### Socioeconomic variables

Municipal financial statements are declared annually, making it possible to identify expenses incurred in different sectors of Education [26]. In the present analysis, we have used the total amount spent on all educational levels, since many schools have more than one educational cycle or may offer other teaching modalities not included in this analysis. Thus, the final amount spent on education is indirectly associated with the schools’ educational performances.

The value of all elementary and high school teachers’ salaries were selected by the respective Annual List of Social Information microdata occupation codes [27]. The extreme values were removed after the z-score transformation and elimination of observations with more than 3 standard deviations, with the average wages being calculated for each municipality.

The fraction of the municipal GDP destined to Education is part of a category that comprises the following government sectors: administration, defense, education, public health and social security. The level of aggregation disclosed does not allow for a greater degree of refinement, which would make it possible to identify the budget for each school. Educational GDP per capita was calculated using the population estimate by the Brazilian Institute of Geography and Statistics (IBGE).

### Model

**Fig 1:**
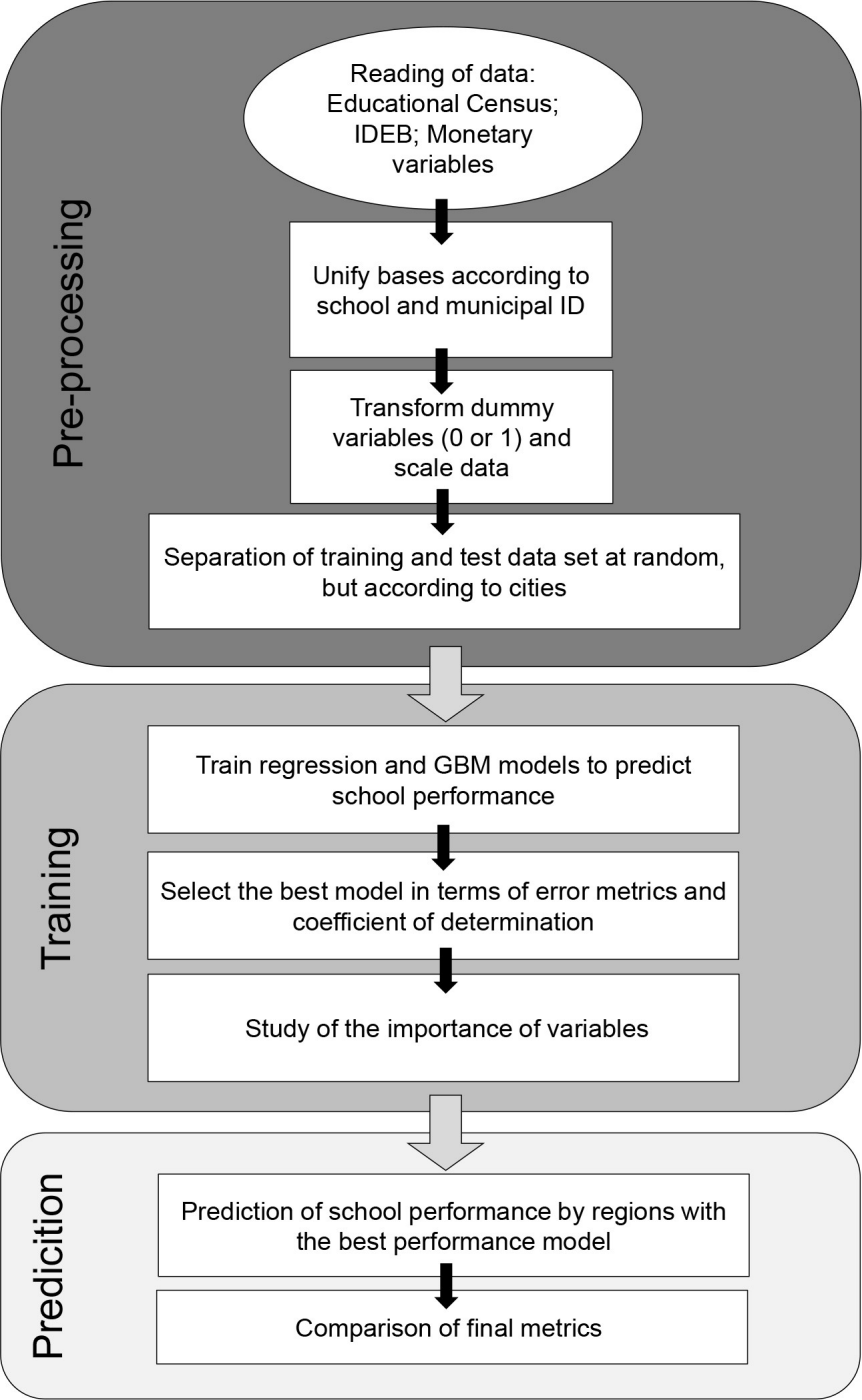
Flowchart illustrating the procedures and steps performed. GBM: Gradient Boosting Machine; IDEB: Basic Education Development Index.

All data sets were loaded and unified according to the school or city code and data were pre-processed. Subsequently, the models were trained, and predictions were made. The error metrics and the determination coefficient were calculated for each of the models in the respective school cycles. With these statistics, the best predictive performance was chosen to study the importance of variables. Finally, IDEB predictions were made according with the five geopolitical regions of Brazil, which constitute well-established spatial groupings (North, Northeast, Midwest, South and Southeast), each with specific characteristics (climate, economy, demographic composition, among others). Fig 1 summarizes the main steps carried out in this analysis.

### Pre-processing

The public schools were the focus of this analysis (state and municipal schools), since they concentrate 80% of the students. The data were segmented into three main data sets corresponding to the educational cycles analyzed: Early Years of Elementary School (1^st^ - 5^th^ grade), Final Years of Elementary School (6^th^ - 9^th^ grade) and High School (10^th^ - 12^th^ grade). Each set was divided according with the municipalities where the schools were located, this strategy aimed to reduce the possible effects of spatial correlation.

Thus, a random sample of Brazilian cities for each educational cycle was selected. Subsequently, they were divided into two subsets, with the proportion of schools approximately 70% for the training data and 30% for the test data. Predictive models were built using only the training data and, after consolidating them, predictions for the IDEB were made with the test data. The performance of the models was optimized through cross-validation during training, before implementing them in the test subset. This strategy allowed the performance of the algorithms to have a similar result for the training data and the test data.

Categorical variables went through the “one ‑ hot encoding” process, where they were transformed into dummy variables and one of the categories in the set of dummy variables was removed, a condition that reduced the collinearity between the factors. After these processes the data set consisted of 61 variables. To maximize the performance of the algorithms, the data were placed on the same scale, where all continuous variables were normalized by the z-score. The mean and standard deviation values obtained for the training set variables were applied to the scale transformation of the test set. The performance of the different models was evaluated by the error metrics and by the coefficient of determination being the best model selected for the study of the importance of variables.

### Methods for prediction methods and variable importance

To analyze the association between school performance and other descriptive variables, multiple linear regression models, penalized regressions and the GBM [28, 29] were generated to predict the IDEB of each school. Linear regression is a linear approach widely used to investigate the association between a set of predictor variables to a response variable (IDEB).

The penalized regressions used three different algorithms: Lasso, Ridge and Elastic Net, common techniques for reducing the complexity of the data set [30]. The LASSO regression (Least Absolute Shrinkage and Selection Operator) is an optimization in which the coefficients are penalized by an absolute value, the sum of the estimators, and minimized by the quadratic error, when there are variables with a high degree of correlation between them. This method eliminates variables from the analysis, due to high penalties, which causes a reduction in the dimensionality of the model. Ridge Regression reduces the effect of variables also by penalties, adding a bias to the model that generates restrictions to the coefficients without zeroing them [30]. The elastic net is a linear combination of Ridge and LASSO penalties, with a parameter defined as α and ranging from 0 to 1. Thus, if α = 0, the penalty function will reduce the term referring to Ridge, if α = 1 the term penalized will be LASSO. Therefore, values between 0 and 1 make the elastic net. For this study, the value 0.5 was assumed, being an option to supply restrictions of LASSO (dependence of the data set) and Ridge (susceptible to high variance), where some coefficients will be eliminated and the selection will become more sparse [30].

The gradient improves the regression model and the GBM is a tool used to capture complex dependencies of nonlinear functions using regression trees, which are unidirectional graphs, formed by nodes and branches. The decision nodes imply logical tests which, through their responses—the branches, successively direct the data set to the final node, where there is no choice to be made and the answer is obtained [23].

Learning occurs by combining parameters for the model in which the loss function is minimized for a given set of training data samples and their corresponding destinations. The purpose of the loss function in GBM is to penalize large deviations from the desired results, in addition to neglecting small residues. The algorithm of this model requires: (i) a loss or cost function, which can be: the mean absolute error; the sum of the quadratic errors or the quadratic error; and (ii) a weak learner optimizer [31]. All models were generated using 1000 trees, considering 10 depth interactions with a learning rate of 0.01.

The choice of the best model was made in terms of the coefficient of determination and the error metrics calculated for all models. The mean squared error (MSE) calculates the square difference between the observed value and the value predicted by the model, after which the mean of the differences is calculated. The root mean square error (RMSE) consists of the square root of the MSE, which leaves the errors on the same original scale, whereas the mean absolute error (MAE) measures the mean magnitude of errors for the set of predictions, without considering the direction of the differences. The higher the value of any of the errors, the worse the performance of the model. The determination coefficient (R^2^) was calculated as the correlation between the values performed and the values predicted squared.

## Results

### Descriptive statistics

The descriptive analysis of IDEB shows that the average value is different in each educational cycle, as school complexity increases, the evaluation grades gradually fall. The worst performance was in high school, regardless of the region. Since the beginning of the IDEB implementation in 2005, there have been improvements in national averages, but this progress has been slow and differ for each school cycle. In twelve years of evaluation, the classes analyzed in the Early Years of Elementary Education increased 2.15 points, going from 3.65 to 5.8, configuring the educational cycle with the best performance. The Final Years of Elementary School, for an equal period of comparison, increased 1.2 points, and in the first assessment, the national results for public schools were 3.2. Secondary Education started from 3 points reaching the level of 3.5, showing that since the IDEB implementation, this cycle has progressed only 0.5 points (S2 Fig).

In a slightly more detailed investigation, individual school performance was monitored for each exam (since 2005), to identify whether grades improved, maintained or decreased in their performance, and afterwards the absolute number for each category was computed. In general, there has been little improvement in performance and between 6% to 7% of schools showed results equal to the immediately previous assessment. Regarding the decrease in grades, for the last period (2015–2017), 30.5% of schools had a reduction in grades, while 62.5% of schools managed to improve their exam performance S3 Fig.

### Comparison of models

These results allowed an initial perspective to the national scenario, in which all schools were analyzed simultaneously, without any distinction of region. The method that obtained the best performance was GBM, with its error metrics lower than the other models and the determination coefficient with the best performance. These findings indicate greater predictive capacity, when compared to other methods, a condition that suggested the absence of linearity for the analyzed data sets. When correlating the data performed with those predicted, a moderate correlation between the data (r_s_ = 0.5–0.6, p < 0.05) was identified. The regressions obtained similar results, but with results lower than those in Table 1.

**Table 1:** Error metrics for the analyzed models.

		Multiple Regression		GBM		LASSO		Ridge		Elnet	
		Train	Test	Train	Test	Train	Test	Train	Test	Train	Test
Early Years	MSE	0.658	0.634	0.538	0.579	0.658	0.634	0.659	0.634	0.658	0.634
Final Years	MSE	0.792	0.760	0.645	0.683	0.792	0.761	0.793	0.761	0.792	0.761
High School	MSE	0.624	0.701	0.525	0.582	0.636	0.696	0.634	0.697	0.635	0.696
Early Years	RMSE	0.811	0.796	0.733	0.761	0.811	0.796	0.812	0.796	0.811	0.796
Final Years	RMSE	0.890	0.872	0.803	0.826	0.890	0.872	0.890	0.872	0.890	0.872
High School	RMSE	0.790	0.837	0.725	0.763	0.797	0.835	0.796	0.835	0.797	0.834
Early Years	MAE	0.638	0.622	0.577	0.593	0.638	0.622	0.639	0.622	0.638	0.622
Final Years	MAE	0.701	0.686	0.632	0.647	0.701	0.686	0.701	0.687	0.701	0.686
High School	MAE	0.626	0.646	0.570	0.590	0.632	0.648	0.630	0.648	0.632	0.648
Early Years	R^2^	0.342	0.365	0.466	0.421	0.342	0.366	0.341	0.367	0.342	0.366
Final Years	R^2^	0.208	0.226	0.361	0.305	0.208	0.226	0.207	0.226	0.208	0.226
High School	R^2^	0.376	0.348	0.475	0.459	0.365	0.351	0.369	0.352	0.366	0.352

GBM: Gradient Boosting Machine.

When comparing the training and test values, for all models, we identified that no model had high specificity, therefore the training of the model did not generate overfitting, since there was no decrease in performance with the estimation performed with the set of tests. Regardless of the technique used, Final Years data always showed worse performance compared with other school cycles.

### Importance of variables

**Fig 2:**
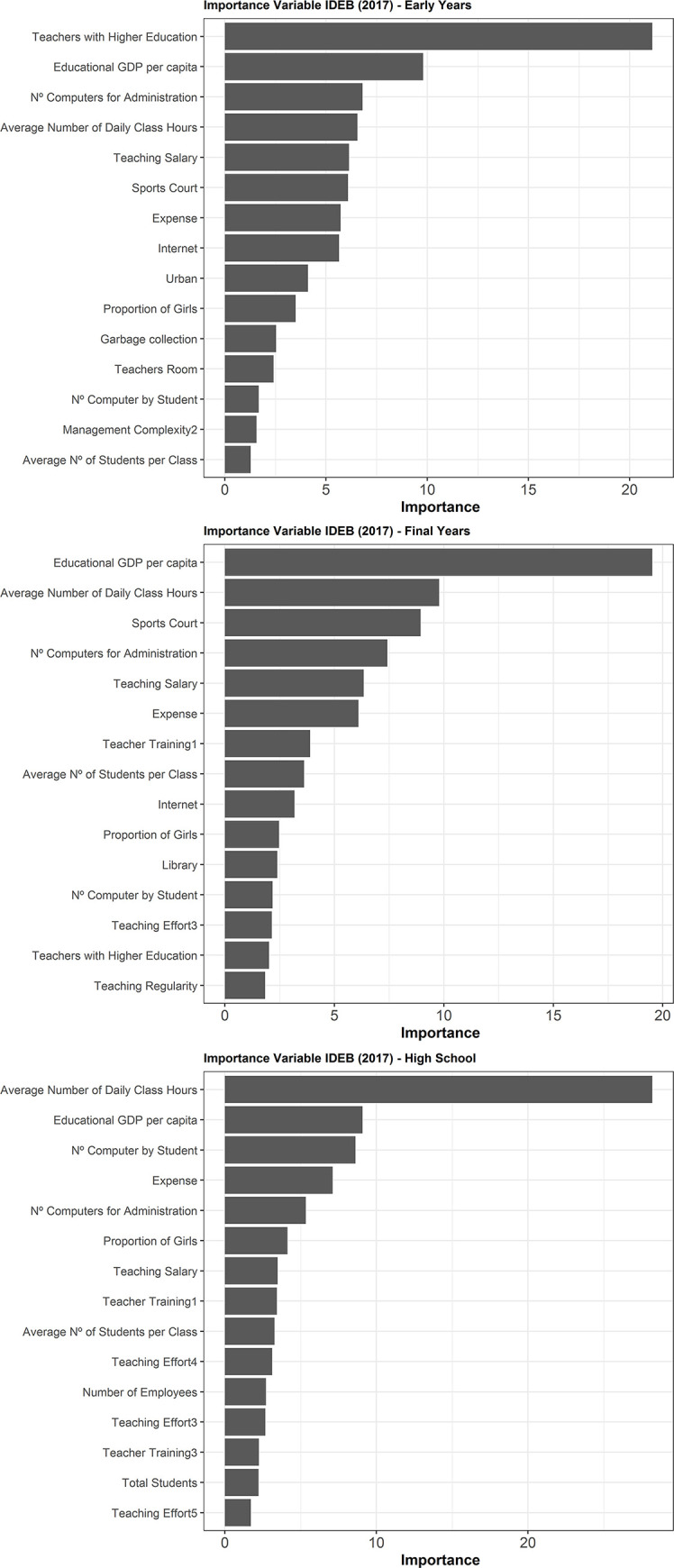
Importance of variables calculated using the GBM for the Early Years, Final Years and High School. IDEB: Basic Education Development Index; GDP: Gross Domestic Product.

The model generated by the GBM obtained the best predictive result, hence it was used to study the importance of the variables. We found that the most relevant factors varied for each educational phase, reflecting the innumerable particularities for each period Fig 2. We observed that monetary variables had relevance in the constitution of IDEB for all models, however, one of the main objectives of this analysis was to identify factors not directly linked to investment.

For Early Years, the most important variable was the rate of teacher with higher education, indicating the relationship between teacher training and school performance. Subsequently, the greatest relevance was the educational GDP per capita, detecting the association between the investment grade and school performance, followed by the number of computers destined for school administration.

The main descriptors of the Final Years were the GDP per capita, the average number of daily classes and the presence of courts at school, whereas High School was better described by the average number of daily classes, GDP per capita and the number of computers per student.

### Correlation of the importance of variables

To assess how different the importance of variables was between educational cycles, Spearman correlations were calculated pairwise. All relationships were significant (p < 0.01). The magnitude of the correlations is associated with how close the school cycles are: Early Years x Final Years (r_s_ = 0.808), Final Years x High School (r_s_ = 0.739) Early Years x High School (r_s_ = 0.533).

### Regional difference

The same parameters used in the implementation of the GBM for data from Brazil were used to generate the predictions of the regional IDEB, and we found particularities for each one of them and their respective school cycles. In comparison with the country level, there was variation in terms of the coefficient of determination, with most regions showing a lower performance for this indicator, considering the data used in the test set. The error metrics had less variation between training and testing, but compared with the general model, there was greater variation for the Initial and Final Years cycles Table 2.

**Table 2:** Metrics for the GBM in all Brazilian regions, considering the different educational cycles.

Cycles	Metrics	Country	Midwest	North	Northeast	South	Southeast
Early Years	MAE	0.593	0.557	0.596	0.733	0.516	0.611
Final Years		0.647	0.675	0.537	0.696	0.590	0.672
High School		0.590	0.697	0.608	0.575	0.553	0.596
Early Years	MSE	0.579	0.498	0.563	0.859	0.429	0.613
Final Years		0.683	0.719	0.482	0.797	0.553	0.735
High School		0.582	0.719	0.584	0.538	0.542	0.596
Early Years	RMSE	0.761	0.706	0.750	0.927	0.655	0.783
Final Years		0.826	0.848	0.694	0.893	0.744	0.858
High School		0.763	0.848	0.764	0.734	0.736	0.772
Early Years	R^2^	0.421	0.091	0.549	0.283	0.084	0.370
Final Years		0.305	0.075	0.436	0.252	0.141	0.165
High School		0.459	0.369	0.265	0.593	0.476	0.370

Due to the variance found between the regions, the inherent heterogeneity of the regions was emphasized, indicating that there is a need for further investigation to detect possible patterns Table 2.

School performance, measured by IDEB, was reduced over the educational cycles in all regions. The socioeconomic variables were frequently found to be the most relevant predictor to the performance of elementary education, with expenditure, educational GDP per capita and teaching salary being respectively the elements with the greatest impact on IDEB. The average number of students and the average number of daily classes were discretionary components of IDEB present in all cycles, together it was detected as a relevant condition in the constitution of IDEB. In a comparative analysis between states in the southeast region, a similar pattern was identified between states S4–S6 Figs and S8 Table.

## Discussion

In this work, we aimed at identifying factors that could be possibly related to school performance, at national and regional levels for the three Brazilian educational cycles. For that, different types of regressions and a machine learning method (GBM) were used to analyze a data set based on educational and economic research. After evaluating the models, carried out in terms of error metrics and coefficients of determination, the importance of each variable for school performance was identified for the GBM method, which obtained the best predictive performance, and later IDEB regional predictions were made.

The overall performance of schools in IDEB improved since its implementation, for all school cycles, but if individual school follow-up is observed, most schools do not assume a performance improvement path. This way, IDEB performance of approximately 62% of schools, regardless of the educational cycle, remains the same compared with the performance of the previous period. Only 7% shows better performance compared with the previous year. This finding indicates that in-depth investigations must be carried out to better understand the elements that cause the increase in global scores.

The regression methods did not achieve the same performance as the GBM. One of the possible reasons for the best performance of the GBM model is the absence of rigid assumptions, among them the assumption of non-linearity between the elements analyzed, or even its high modeling and forecasting accuracy of response variables [28]. One of the main elements for school performance is the socioeconomic status of students [10, 32], captured here from GDP per capita. Despite this variable generalizing income by municipality, for all school cycles it was among the three factors of greatest contribution to forecasting IDEB Fig 2. Some studies reinforce the non-linearity between income and school performance, because at the microeconomic level, family income per capita is not linearly associated with other variables [33].

Regarding the elements associated with IDEB for each educational cycle, we identified that the factors of greatest contribution are different for each cycle. Thus, a possibility for high school, a cycle with lower performance, it is crucial to consider some measures (e.g., increasing hours of classes) during the formulation of public policies, that may not have the same effectiveness and efficiency for another school cycle, even if there is a strong correlation between some cycles.

The difference between educational cycles and between regions was found, reflecting the historical regional difference well known in Brazil [12, 34]: North and Northeast were the regions with the worst performance, regardless of the school level analyzed. This result is not new, but rather persistent. In the past, the differences used to come from high illiteracy rates and lower enrollment rates, now these two regions have the lowest school performance, according to IDEB. The lack of convergence of performance between regions indicates that policies carried out at the national level are not able to obtain the same results for all regions. This encourages investigations on specific needs, with subsequent design of focal policies to make school performance less heterogeneous and, consequently, reduce social inequalities.

Public policies for education should consider the effect of variables on school performance between school cycles and regions, so that the implementation of new practices could potentially maximize student’s performance. The results indicate a decrease in the performance of the model in some regions, and the impact of the variables is not the same for all regions. For late years of elementary school, the Midwest region had the worst performance, and as the variable with the greatest effect is GDP per capita, it may be that the increase in this variable has no impact on the quality of education.

Educational heterogeneity is found in several countries in Latin American [35] and the search for a homogeneous system is genuine. Therefore, the present research can be seen as a case study, since its findings may be similar to those of other educational systems in countries with similar economic situation or with disjunct school structure.

According with the structure of each region, it was already expected that for the same model there would be uneven performances, a condition related to the historical and economic conditions of those places, which currently manifest themselves in terms of human capital. Thus, it was possible to detect that the same model does not have the same predictive capacity for all regions and school cycles, indicating that other factors not incorporated in this analysis may interfere in the predictive capacity of the future IDEB. This limitation can be reduced by adding new variables or by refining the data. As for the variables, it is known that the school failure rate, indicated by countless studies, is one of the main conditions for future school performance. However, at this moment it is relevant to identify the existence of factors for more immediate action, such as increasing the number of classes or reducing the average number of students per classroom so that positive results can come sooner.

In addition to deepening in other Machine Learning methods, there are some investigations that were not privileged at this time, as our main objective was to identify the feasibility of using new techniques to assess the factors that contribute to good educational performance. We suggest future work could refine the parameters of the GBM, which obtained the best performance, as well as incorporate other years of evaluation, consolidating a longitudinal approach. Another possibility is to insert other descriptors of the school structure, composition of the classrooms and other economic variables. However, it is noteworthy that it was already possible to identify that school performance is better described by non-linear methods and that heterogeneity is present in the most relevant factors in educational and regional levels.

Similar studies already report the efficiency of Machine Learning methods in predicting school performance, however, few studies have used IDEB in more comprehensive analyzes that allow simultaneous comparison between educational cycles and Brazilian geopolitical regions. In general, the most used models are those of classification, in order to detect whether students will pass, whether they will abandon the school year, and other issues, for very specific regions such as cities or even the number of classes. The identification of new elements that help in the prediction of IDEB, can be an alternative for the design of new educational policies. Considering the current scenario of economic restriction, it is possible to prioritize the elements that are not directly linked to educational expenses in a fixed and continuous way, but those of a specific character and temporarily delimited.

Non-linear methods have greater predictive capacity for school performance and constitute a methodology to be explored in Brazil, where only few studies are available [36] opposing to the world scenario, where such tools have been widely used to detect factors related to school performance by many ways [24, 37–40]. Educational data can assist in the formation of tools for educational monitoring, as is already the case in the environmental area [41]. The use of Data Science has proven to be a form of differential analysis, which has allowed companies to maximize their productivity and profits. Data-based decision-making makes resource management more effective and can assist public managers [42].

This work brings new aspects about the performance evaluation of public schools, when considering the analyzes of the three educational periods of Brazilian basic education. We detected which are the most relevant factors for each of the educational cycles, and they should be considered for future educational policies. Those factors directly or indirectly related to school performance should be considered by policy makers who should consider the impact of specific conditions on the composition of local policies, given the heterogeneous character of the school performance. In conclusion, we believe that the proposed approach, based on non-linear models, is a promising tool to obtain new perspectives for the association of variables that describe school structure and school performance, in addition to allowing the detection of the elements that have the greatest influence on school performance.

## Supporting information

S1 Fig IDEB 2017 boxplots for the different educational cycles Early Years, Final Years and High School and Brazilian regions. (DOCX)

S2 Fig Historical IDEB for the different educational cycles Early Years, Final Years and High School. (DOCX)

S3 Fig Evaluation of school performance in relation to the result of the previous IDEB since 2005. (DOCX)

S4 Fig Importance of variables calculated using the GBM for the Early Years in southeastern states. (DOCX)

S5 Fig Importance of variables calculated using the GBM for the Final Years in southeastern states. (DOCX)

S6 Fig Importance of variables calculated using the GBM for the High School in southeastern states. (DOCX)

S1 Table Descriptive statistics for the IDEB of state and municipal schools, by region. (DOCX)

S2 Table Proportion of schools in the early years by region and states considering the categorical variables that describe the school structure. (DOCX)

S3 Table Proportion of schools in the early years by region and states considering the categorical variables that describe the school structure (continued). (DOCX)

S4 Table Proportion of schools in the final years by region and states considering the categorical variables that describe the school structure. (DOCX)

S5 Table Proportion of schools in the final years by region and states considering the categorical variables that describe the school structure (continued). (DOCX)

S6 Table Proportion of schools in the high school by region and states considering the categorical variables that describe the school structure. (DOCX)

S7 Table Proportion of schools in the high school by region and states considering the categorical variables that describe the school structure (continued). (DOCX)

S8 Table Metrics for the GBM in all states of southeastern, considering the different educational cycles. (DOCX)
