# Supplementary material for: Assessing the educational performance of different Brazilian school cycles using data science methods
Source: PLoS One. 2021 Mar 17;16(3):e0248525. doi: 10.1371/journal.pone.0248525 (PMC7968699; doi:10.1371/journal.pone.0248525)
Supplement: S1 Fig — (DOCX) [file pone.0248525.s001.docx]

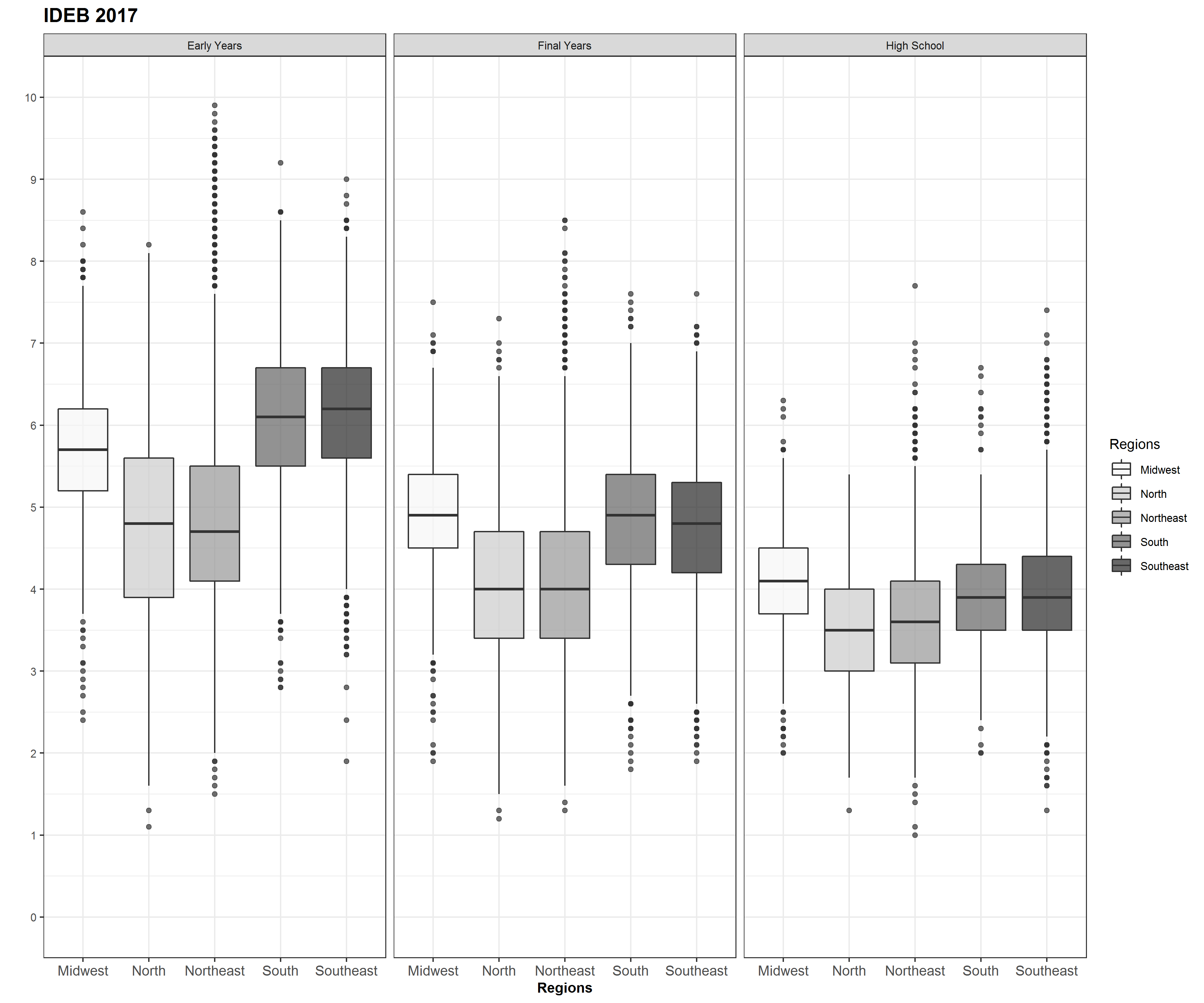


**S1 Fig. IDEB 2017 boxplots for the different educational cycles - Early Years, Final Years and High School and Brazilian regions.**

IDEB: Basic Education Development Index.
