# Supplementary figures and images for: Assessing the educational performance of different Brazilian school cycles using data science methods
Source: PLoS One. 2021 Mar 17;16(3):e0248525. doi: 10.1371/journal.pone.0248525 (PMC7968699; doi:10.1371/journal.pone.0248525)

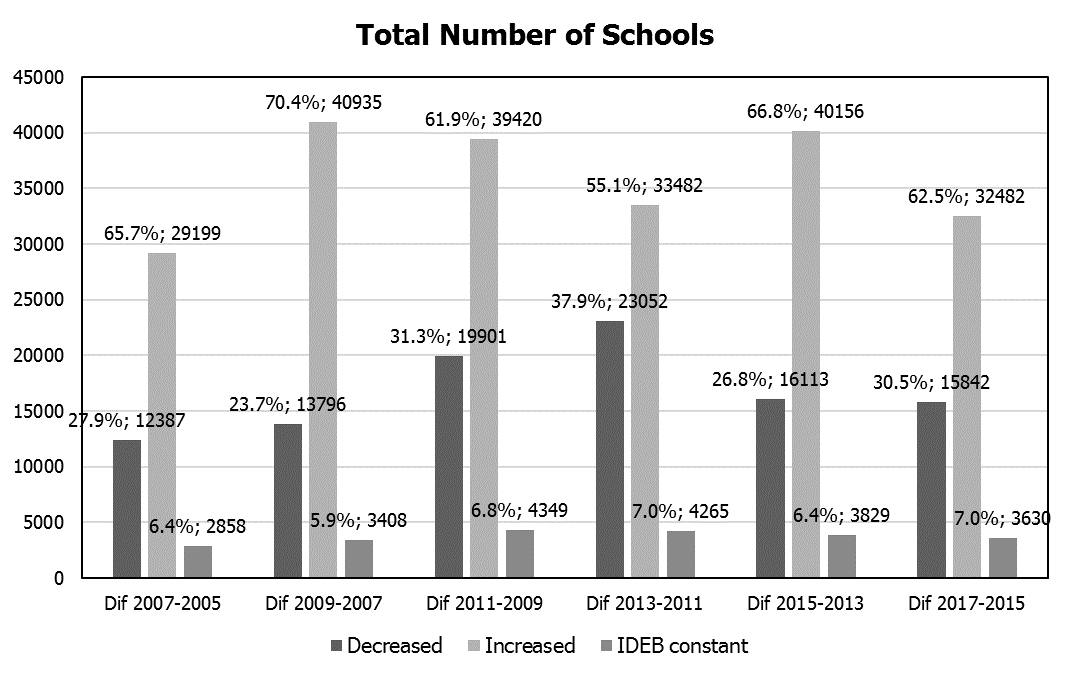


**S3 Fig. Evaluation of school performance in relation to the result of the previous IDEB, since 2005.**

Supplement: S3 Fig — (DOCX) [file pone.0248525.s003.docx]
