# Supplementary material for: Assessing the educational performance of different Brazilian school cycles using data science methods
Source: PLoS One. 2021 Mar 17;16(3):e0248525. doi: 10.1371/journal.pone.0248525 (PMC7968699; doi:10.1371/journal.pone.0248525)
Supplement: S4 Fig — (DOCX) [file pone.0248525.s004.docx]

**
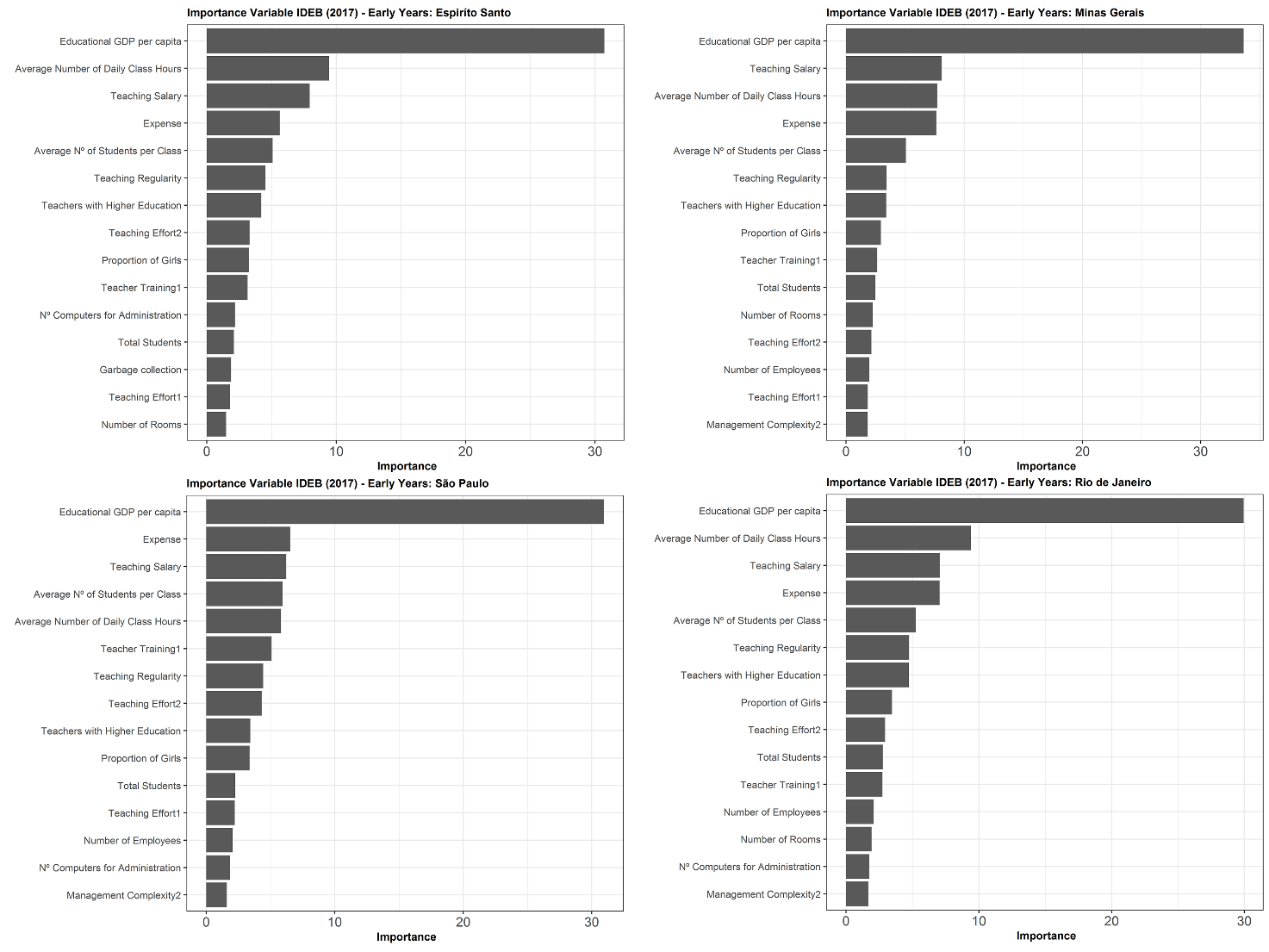
**

**S4 Fig. Importance of variables calculated using the GBM for the Early Years in southeastern states.**

IDEB: Basic Education Development Index; GBM: Gradient Boosting Machine; GDP: Gross Domestic Product.
