# Supplementary material for: Assessing the educational performance of different Brazilian school cycles using data science methods
Source: PLoS One. 2021 Mar 17;16(3):e0248525. doi: 10.1371/journal.pone.0248525 (PMC7968699; doi:10.1371/journal.pone.0248525)
Supplement: S1 Table — (DOCX) [file pone.0248525.s007.docx]

**S1 Table. Descriptive statistics for the IDEB of state and municipal schools, by region.**

|  | Early Years | | | | | | Final Years | | | | | | | High School | | | | | |
| --- | --- | --- | --- | --- | --- | --- | --- | --- | --- | --- | --- | --- | --- | --- | --- | --- | --- | --- | --- |
|  | **Country** | **Midwest** | **North** | **Northwest** | **South** | **Southeast** | **Country** | **Midwest** | **North** | **Northwest** | **South** | **Southeast** | **Country** | | **Midwest** | **North** | **Northwest** | **South** | **Southeast** |
| **N** | 41,515 | 2,921 | 4,478 | 14,224 | 6,803 | 13,089 | 25,530 | 1,900 | 2,672 | 9,088 | 4,026 | 7,844 | 9,597 | | 789 | 693 | 3,055 | 987 | 4,073 |
| Max | 9.9 | 8.6 | 8.3 | 9.9 | 9.2 | 9.0 | 8.5 | 7.5 | 7.5 | 8.5 | 7.7 | 7.8 | 8.4 | | 8.3 | 7.9 | 8.2 | 7.3 | 8.4 |
| Min | 1.1 | 2.4 | 1.1 | 1.5 | 2.8 | 1.9 | 0.9 | 1.9 | 0.9 | 1.1 | 1.8 | 1.9 | 1.0 | | 2.0 | 1.3 | 1.0 | 1.7 | 1.3 |
| Amplitude | 8.8 | 6.2 | 7.2 | 8.4 | 6.4 | 7.1 | 7.6 | 5.6 | 6.6 | 7.4 | 5.9 | 5.9 | 7.4 | | 6.3 | 6.6 | 7.2 | 5.6 | 7.1 |
| Mean | 5.5 | 5.7 | 4.7 | 4.8 | 6.1 | 6.1 | 4.4 | 4.9 | 4.0 | 4.0 | 4.8 | 4.7 | 4.0 | | 4.3 | 3.8 | 3.8 | 4.1 | 4.2 |
| Median | 5.6 | 5.7 | 4.7 | 4.7 | 6.1 | 6.2 | 4.5 | 4.9 | 4.0 | 4.0 | 4.9 | 4.8 | 3.9 | | 4.2 | 3.6 | 3.6 | 4.0 | 4.0 |
| Mode | 6.1 | 5.9 | 4.4 | 4.6 | 6.1 | 6.3 | 4.6 | 4.9 | 4.0 | 4.0 | 5.0 | 4.8 | 3.9 | | 4.5 | 3.5 | 3.4 | 3.7 | 3.9 |
| Variance | 1.3 | 0.6 | 1.3 | 1.2 | 0.7 | 0.7 | 0.9 | 0.5 | 0.8 | 0.9 | 0.6 | 0.6 | 1.0 | | 0.8 | 1.0 | 0.9 | 0.8 | 0.9 |
| Standard Deviation | 1.2 | 0.8 | 1.1 | 1.1 | 0.8 | 0.8 | 0.9 | 0.7 | 0.9 | 0.9 | 0.8 | 0.8 | 1.0 | | 0.9 | 1.0 | 1.0 | 0.9 | 1.0 |
| 1st Quarter | 4.7 | 5.2 | 3.9 | 4.0 | 5.5 | 5.6 | 3.8 | 4.4 | 3.4 | 3.4 | 4.3 | 4.2 | 3.4 | | 3.8 | 3.1 | 3.1 | 3.5 | 3.6 |
| 2nd Quarter | 5.6 | 5.7 | 4.7 | 4.7 | 6.1 | 6.2 | 4.5 | 4.9 | 4.0 | 4.0 | 4.9 | 4.8 | 3.9 | | 4.2 | 3.6 | 3.6 | 4.0 | 4.0 |
| 3rd Quarter | 6.3 | 6.2 | 5.5 | 5.4 | 6.6 | 6.7 | 5.1 | 5.4 | 4.6 | 4.6 | 5.3 | 5.3 | 4.5 | | 4.7 | 4.3 | 4.3 | 4.5 | 4.6 |
| 4th Quarter | 9.9 | 8.6 | 8.3 | 9.9 | 9.2 | 9.0 | 8.5 | 7.5 | 7.5 | 8.5 | 7.7 | 7.8 | 8.4 | | 8.3 | 7.9 | 8.2 | 7.3 | 8.4 |

IDEB: Basic Education Development Index.
