# Supplementary material for: Assessing the educational performance of different Brazilian school cycles using data science methods
Source: PLoS One. 2021 Mar 17;16(3):e0248525. doi: 10.1371/journal.pone.0248525 (PMC7968699; doi:10.1371/journal.pone.0248525)
Supplement: S3 Table — (DOCX) [file pone.0248525.s009.docx]

**S3 Table. Proportion of schools in the early years by region and states, considering the categorical variables that describe the school structure. The color of each cell indicates the presence of the resource, based on the percentage of the schools that own such resource. Green for high presence of the resource, yellow for moderate presence, and red for weak presence (continued).**

| **Unity** | **Management Complexity1** | **Management Complexity2** | **Management Complexity3** | **Management Complexity4** | **Management Complexity5** | **Management Complexity6** | **Municipal** | **Refectory** | **Science Lab** | **Secretary** | **Sewer** | **Special needs** | **Sports Court** | **Teachers Room** | **Printer** | **TV** | **Urban** | **Water** |
| --- | --- | --- | --- | --- | --- | --- | --- | --- | --- | --- | --- | --- | --- | --- | --- | --- | --- | --- |
| Country | 5.0 | 29.9 | 32.0 | 10.2 | 15.3 | 7.6 | 82.5 | 49.8 | 8.2 | 82.6 | 99.2 | 41.5 | 55.2 | 77.6 | 81.5 | 93.9 | 75.4 | 98.8 |
| Midwest | 5.6 | 21.0 | 36.4 | 16.2 | 10.6 | 10.3 | 72.4 | 32.3 | 7.9 | 92.8 | 99.9 | 54.1 | 69.4 | 92.2 | 88.4 | 96.0 | 86.1 | 99.6 |
| Goiás | 9.8 | 32.0 | 34.0 | 8.4 | 11.4 | 4.4 | 87.3 | 12.8 | 4.1 | 91.8 | 99.9 | 55.1 | 54.9 | 88.6 | 88.4 | 96.7 | 90.5 | 99.7 |
| Mato Grosso | 2.8 | 13.0 | 47.7 | 17.2 | 8.1 | 11.2 | 57.4 | 61.9 | 5.4 | 90.4 | 99.7 | 45.7 | 73.0 | 92.8 | 84.3 | 93.9 | 79.1 | 99.0 |
| Mato Grosso do Sul | 1.4 | 10.1 | 27.2 | 29.3 | 12.0 | 20.0 | 63.1 | 32.6 | 18.1 | 97.7 | 100.0 | 62.5 | 91.6 | 98.1 | 93.2 | 97.2 | 86.2 | 100.0 |
| North | 2.1 | 34.0 | 30.6 | 7.2 | 17.6 | 8.5 | 79.1 | 52.0 | 4.4 | 83.3 | 97.5 | 30.3 | 39.6 | 70.3 | 78.0 | 86.2 | 64.4 | 98.4 |
| Acre | 2.7 | 36.3 | 18.7 | 6.6 | 30.8 | 4.9 | 52.2 | 70.9 | 0.5 | 85.2 | 96.2 | 45.6 | 23.1 | 78.0 | 87.4 | 91.2 | 71.4 | 93.4 |
| Amapá | 2.0 | 41.6 | 21.5 | 6.0 | 24.2 | 4.7 | 52.3 | 76.5 | 2.7 | 87.2 | 97.3 | 37.6 | 26.8 | 53.7 | 81.2 | 87.2 | 65.8 | 99.3 |
| Amazonas | 2.1 | 33.7 | 27.1 | 9.1 | 18.1 | 10.0 | 71.1 | 63.0 | 11.4 | 84.8 | 95.9 | 11.8 | 30.5 | 73.6 | 80.0 | 83.8 | 72.9 | 99.3 |
| Pará | 1.2 | 33.9 | 30.1 | 6.4 | 18.4 | 10.0 | 89.8 | 46.8 | 1.9 | 81.0 | 97.2 | 30.0 | 39.9 | 60.6 | 70.3 | 84.0 | 56.7 | 97.9 |
| Rondônia | 2.5 | 30.8 | 42.4 | 6.5 | 10.6 | 7.2 | 72.5 | 61.8 | 5.3 | 81.0 | 99.8 | 45.1 | 57.6 | 89.6 | 90.3 | 92.6 | 63.2 | 99.1 |
| Roraima | 5.3 | 50.0 | 32.9 | 0.0 | 9.2 | 2.6 | 100.0 | 78.9 | 1.3 | 85.5 | 100.0 | 39.5 | 50.0 | 76.3 | 92.1 | 89.5 | 60.5 | 100.0 |
| Tocantins | 5.2 | 31.7 | 35.5 | 9.9 | 13.2 | 4.4 | 74.4 | 15.4 | 2.5 | 90.6 | 99.7 | 43.0 | 49.0 | 87.6 | 86.0 | 90.9 | 80.2 | 100.0 |
| Northwest | 4.2 | 28.6 | 28.2 | 8.9 | 22.5 | 7.6 | 95.4 | 18.1 | 2.2 | 68.9 | 98.7 | 36.0 | 28.2 | 55.4 | 72.1 | 91.2 | 58.3 | 97.0 |
| Alagoas | 2.7 | 29.6 | 14.6 | 10.1 | 30.2 | 12.9 | 94.7 | 18.7 | 2.7 | 74.4 | 99.8 | 39.0 | 23.4 | 61.6 | 69.0 | 94.3 | 55.5 | 99.4 |
| Bahia | 6.4 | 40.9 | 20.3 | 8.2 | 20.0 | 4.1 | 99.6 | 16.5 | 1.3 | 68.8 | 98.9 | 29.4 | 25.6 | 49.5 | 77.0 | 90.7 | 58.1 | 98.3 |
| Ceará | 1.5 | 17.7 | 45.9 | 8.2 | 19.3 | 7.5 | 99.2 | 17.6 | 2.2 | 73.3 | 99.3 | 44.3 | 44.8 | 65.3 | 76.9 | 94.9 | 52.5 | 96.9 |
| Maranhão | 2.4 | 22.2 | 32.5 | 8.1 | 28.0 | 6.9 | 98.8 | 12.5 | 2.0 | 60.5 | 97.4 | 27.9 | 16.6 | 46.8 | 64.0 | 82.3 | 52.3 | 94.9 |
| Paraíba | 6.7 | 25.6 | 14.8 | 12.5 | 30.7 | 9.7 | 83.7 | 24.9 | 4.3 | 61.8 | 99.5 | 39.9 | 26.2 | 55.4 | 64.1 | 94.3 | 70.7 | 96.0 |
| Pernambuco | 5.7 | 27.1 | 23.4 | 8.3 | 22.1 | 13.4 | 99.1 | 21.5 | 2.7 | 80.5 | 99.3 | 32.7 | 24.8 | 58.4 | 69.4 | 91.1 | 65.5 | 97.1 |
| Piauí | 4.7 | 28.2 | 33.7 | 9.0 | 19.8 | 4.6 | 97.5 | 15.5 | 1.6 | 41.4 | 94.3 | 32.7 | 29.4 | 55.9 | 69.2 | 89.6 | 57.1 | 94.6 |
| Rio Grande do Norte | 4.0 | 38.2 | 24.7 | 10.5 | 18.3 | 4.2 | 77.7 | 23.7 | 4.0 | 78.4 | 99.7 | 50.4 | 23.4 | 61.9 | 76.9 | 94.3 | 69.3 | 99.3 |
| Sergipe | 3.5 | 32.8 | 22.1 | 9.9 | 21.9 | 9.9 | 75.6 | 22.2 | 1.8 | 76.9 | 99.8 | 45.2 | 26.8 | 45.2 | 73.7 | 95.0 | 55.4 | 97.0 |
| South | 6.3 | 24.6 | 40.5 | 13.8 | 8.2 | 6.6 | 76.6 | 69.2 | 18.6 | 86.8 | 99.7 | 58.7 | 77.3 | 93.1 | 87.8 | 96.8 | 84.8 | 99.8 |
| Paraná | 15.1 | 39.8 | 27.4 | 3.8 | 9.9 | 4.1 | 99.9 | 56.8 | 3.9 | 84.0 | 99.8 | 54.8 | 75.6 | 93.5 | 88.4 | 95.6 | 86.9 | 99.9 |
| Rio Grande do Sul | 1.7 | 11.5 | 49.6 | 16.0 | 8.9 | 12.3 | 61.1 | 84.0 | 34.7 | 92.6 | 99.8 | 56.6 | 81.7 | 94.1 | 89.2 | 97.3 | 83.4 | 99.7 |
| Santa Catarina | 1.7 | 23.4 | 44.7 | 23.4 | 5.3 | 1.6 | 68.5 | 64.3 | 14.9 | 82.3 | 99.6 | 66.6 | 73.5 | 91.2 | 84.9 | 97.6 | 83.9 | 100.0 |
| Southeast | 5.8 | 34.2 | 31.2 | 9.5 | 12.0 | 7.4 | 75.7 | 73.7 | 10.2 | 91.7 | 99.7 | 39.3 | 72.8 | 91.0 | 87.5 | 97.0 | 88.9 | 99.9 |
| Espírito Santo | 3.1 | 30.4 | 39.4 | 7.9 | 12.5 | 6.7 | 78.9 | 79.6 | 17.7 | 91.9 | 99.7 | 56.3 | 64.6 | 95.9 | 87.5 | 96.1 | 82.7 | 99.5 |
| Minas Gerais | 6.2 | 25.6 | 31.5 | 13.0 | 12.1 | 11.6 | 63.0 | 74.4 | 13.5 | 88.6 | 99.5 | 47.2 | 72.1 | 89.5 | 91.2 | 97.1 | 84.9 | 99.9 |
| Rio de Janeiro | 4.3 | 31.7 | 35.8 | 11.8 | 8.6 | 7.6 | 99.9 | 93.3 | 9.5 | 92.1 | 99.8 | 50.3 | 54.1 | 81.1 | 84.4 | 97.5 | 85.0 | 99.8 |
| São Paulo | 6.5 | 42.1 | 27.9 | 6.3 | 13.1 | 4.2 | 75.2 | 64.7 | 6.9 | 94.0 | 99.9 | 26.7 | 81.9 | 95.2 | 85.8 | 96.9 | 94.3 | 100.0 |
