# Supplementary material for: Assessing the educational performance of different Brazilian school cycles using data science methods
Source: PLoS One. 2021 Mar 17;16(3):e0248525. doi: 10.1371/journal.pone.0248525 (PMC7968699; doi:10.1371/journal.pone.0248525)
Supplement: S8 Table — (DOCX) [file pone.0248525.s014.docx]

**S8 Table. Metrics for the GBM in all states of southeastern, considering the different educational cycles.**

|  |  | **Early Years** | | **Final Years** | | **High School** | |
| --- | --- | --- | --- | --- | --- | --- | --- |
| State | Statistic | Train | Test | Train | Test | Train | Test |
| Espírito Santo | MAE | 0.590 | 0.606 | 0.623 | 0.727 | 0.544 | 0.571 |
| Minas Gerais | MAE | 0.591 | 0.663 | 0.639 | 0.684 | 0.546 | 0.589 |
| Rio de Janeiro | MAE | 0.596 | 0.678 | 0.636 | 0.665 | 0.542 | 0.591 |
| São Paulo | MAE | 0.600 | 0.677 | 0.631 | 0.682 | 0.551 | 0.604 |
| Espírito Santo | MSE | 0.563 | 0.578 | 0.627 | 0.842 | 0.483 | 0.526 |
| Minas Gerais | MSE | 0.568 | 0.694 | 0.664 | 0.734 | 0.485 | 0.552 |
| Rio de Janeiro | MSE | 0.579 | 0.728 | 0.654 | 0.707 | 0.480 | 0.559 |
| São Paulo | MSE | 0.586 | 0.725 | 0.637 | 0.761 | 0.499 | 0.584 |
| Espírito Santo | R² | 0.439 | 0.294 | 0.375 | 0.177 | 0.517 | 0.495 |
| Minas Gerais | R² | 0.433 | 0.283 | 0.338 | 0.323 | 0.514 | 0.482 |
| Rio de Janeiro | R² | 0.423 | 0.357 | 0.347 | 0.279 | 0.520 | 0.478 |
| São Paulo | R² | 0.416 | 0.322 | 0.364 | 0.218 | 0.501 | 0.482 |
| Espírito Santo | RMSE | 0.750 | 0.760 | 0.792 | 0.918 | 0.695 | 0.725 |
| Minas Gerais | RMSE | 0.754 | 0.833 | 0.815 | 0.857 | 0.697 | 0.743 |
| Rio de Janeiro | RMSE | 0.761 | 0.853 | 0.809 | 0.841 | 0.692 | 0.747 |
| São Paulo | RMSE | 0.765 | 0.852 | 0.798 | 0.872 | 0.706 | 0.764 |

IDEB: Basic Education Development Index; GBM: Gradient Boosting Machine; GDP: Gross Domestic Product. MSE: Mean Squared Error; RMSE: Root Mean Square Error; MAE: Mean Absolute Error; R²: Determination coefficient (R²).
